# Research on Microstructure and Corrosion Behavior of Aluminum Alloy Laser-Welded Joints Assisted by Ultrasonic Vibration

**DOI:** 10.3390/mi16101118

**Published:** 2025-09-29

**Authors:** Di Bai, Ao Li, Jia Liu, Yan Shi, Hong Zhang, Li Yang

**Affiliations:** 1 College of Mechanical and Electric Engineering, Changchun University of Science and Technology, Changchun 130022, China; m19861937022@163.com (A.L.); liujia@cust.edu.cn (J.L.); shiyan@cust.edu.cn (Y.S.); zhanghong@cust.edu.cn (H.Z.); 2 National Base of International Science and Technology Cooperation for Optics, Changchun 130022, China; 3 College of Mechanical and Electrical Engineering, Changchun Polytechnic University, Changchun 130022, China; ylcvit@163.com

**Keywords:** ultrasonic-assisted weld, 6061 aluminum alloy, dendrite refinement, corrosion behavior

## Abstract

Laser welding of 6061 aluminum alloy often results in coarse microstructures and inferior corrosion resistance due to rapid solidification. This study introduces ultrasonic vibration as an auxiliary technique to address these limitations. The paper systematically investigates the influence of laser weld ultrasonic assistance on the microstructure and corrosion behavior of a 6061-T6 aluminum alloy welded joint. The results demonstrate that ultrasonic assistance refined the grain structure and reduced the corrosion current density by 19.1% compared to conventional laser welding, achieving 73.6% of the base metal’s corrosion resistance. The enhancement is attributed to ultrasonic-induced acoustic streaming and cavitation, which promote equiaxed grain formation and impede corrosive penetration. The enhancement is attributed to ultrasonic-induced acoustic streaming and cavitation, which promote equiaxed grain formation and impede corrosive penetration. Under the ultrasonic effect, the number of dimples in the weld fracture increased and the depth was significant, which enhanced the tensile strength of the 6061 Aluminum alloy weld. This work provides a reliable and efficient strategy for producing high-performance aluminum alloy welded structures in industrial applications.

## 1. Introduction

The aluminum alloy was a representative medium-strength material which displayed outstanding plasticity, toughness and corrosion resistance. These advantages enable widespread adoption in lightweight structural design applications [1,2,3,4]. The corrosion resistance of aluminum alloys is intrinsically linked to microstructural characteristics [5]. The welded joint became the most vulnerable to corrosion due to localized microstructural alterations. The laser welding of aluminum alloys offered high processing speed and superior surface morphology [6,7,8].

However, the rapid solidification effect of the molten pool and the redistribution of alloying elements caused significant microstructural modifications in weld zones, which adversely affected corrosion resistance. The ultrasonic-assisted machining technology exhibited significant potential in the regulation of material microstructures [9,10,11,12]. Wang Min et al. [13] investigated the microstructure and mechanical properties of continuous/pulsed hybrid laser shallow penetration welding of 6061 aluminum alloy, demonstrating a notable improvement in weld formation. Lei et al. [14] demonstrated that ultrasonic vibration increased the number of nucleation particles and refined grains, thereby enhancing the mechanical properties of welded joint. Ahmed Teyeb [15] observed that high-intensity ultrasonic treatment effectively disrupted epitaxial dendrite growth. The development of ultrafast laser welding technology demonstrates that spatiotemporal shaping techniques can enable precise manipulation of material microstructures [16]. This provided new research perspectives for further understanding the role of ultrasonic waves in the regulation of molten pool behavior. The ultrasonic vibration primarily generated cavitation effects and acoustic streaming effects during solidification of metal, which induced grain refinement within the metal melt [17,18,19,20].

Most existing studies have primarily focused on the mechanical properties of ultrasonically assisted welded joints, with systematic research on the corrosion behavior and its underlying mechanisms remaining insufficient. This paper investigated the effect of ultrasonic treatment on the microstructure and corrosion behavior of 6061 aluminum alloy welded joints. By applying different ultrasonic powers, the variation law of weld microstructure was analyzed. The mechanism and evolution law of weld corrosion behavior were studied.

## 2. Experimental Procedures

### 2.1. Material

The chemical composition of 6061 aluminum alloy is shown in Table 1. The weld dimensions of the aluminum alloy were 150 mm × 150 mm × 2 mm. Before the welding test, the impurities above the base metal were removed by sandpaper. The surface of the aluminum alloy was cleaned with acetone.

### 2.2. Experimental Equipment and Method

**Figure 1:**
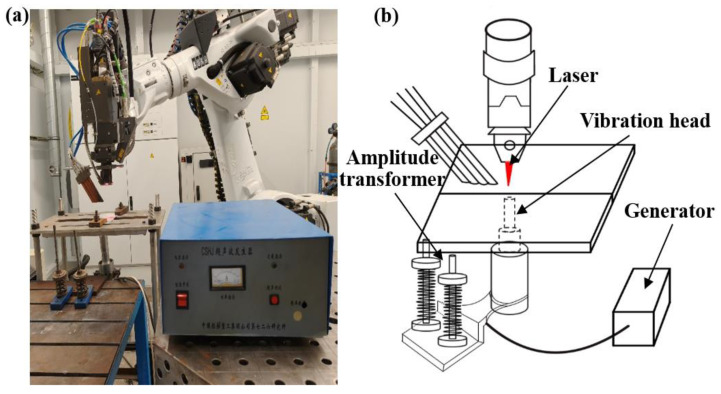
Welding system and ultrasonic device: (**a**) ultrasonic assisted lase welding system; (**b**) schematic diagram of ultrasonic assisted laser welding procedures.

The welding device is shown in Figure 1. The laser welding process was conducted using a Trudisk8002 welding system. The wavelength was 1030 nm. The laser was operated in continuous wave mode. The defocus amount was set to 0.4 mm. The welding speed was 0.03 m/s. The ultrasonic device was composed of a 20 kHz vibration head and an ultrasonic generator. Butt welding was adopted. The amplitude-change pole was connected with the transducer, and the vibration head was in contact with the welding plate bottom. To ensure that the molten pool was subjected to the ultrasonic field throughout its formation and solidification process, the vibration head is positioned vertically at the bottom of the workpiece. The ultrasonic vibration head is fixed by elastic tightening, which reduces the thermal deformation of the welded plate and ensures the stable input of vibration energy. The welding parameters are shown in Table 2.

The metallographic examination samples were prepared by wire cutting. The metallographic etching was carried out by Keller’s reagent (HF:HCl:HNO_3_:H_2_O = 1:1.5:2.5:95). The microstructure was observed by metallographic optical microscope and scanning electron microscope. The tensile tests of each weld joint were conducted, and the fracture morphology was analyzed to evaluate the microstructural features.

### 2.3. Electrochemical Corrosion

**Figure 2:**
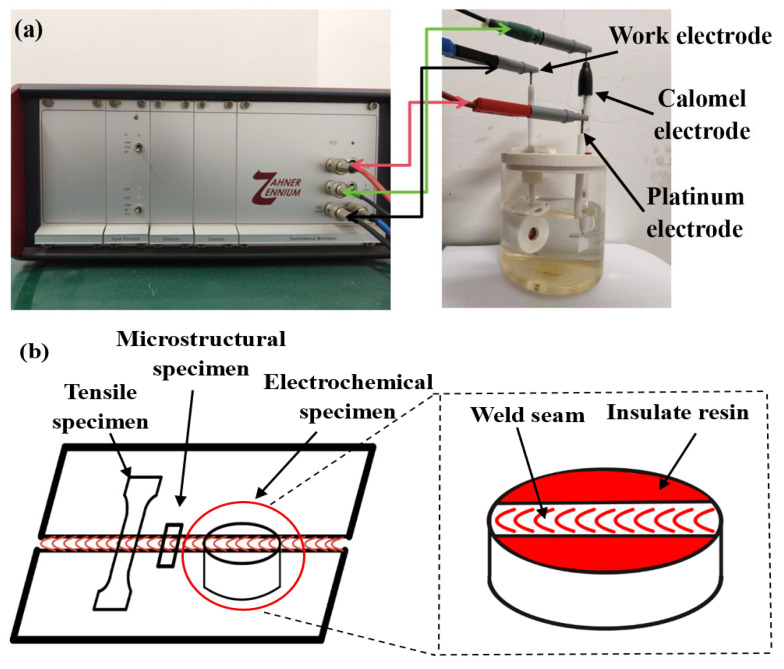
(**a**) Electrochemical laboratory equipment; (**b**) sample preparation method.

The electrochemical workstation and three-electrode system are shown in Figure 2a. The corrosion solution adopted was a 3.5% NaCl solution, and 100% Ar gas was injected for 30 min as a deoxygenation treatment. The exposed area of the working electrode is 0.45 cm^2^, and the remaining areas are covered with insulating resin in Figure 2. The electrochemical impedance spectra were collected. The dynamic polarization curves were collected within ±1 V relative to the OCP at a scanning rate of 2 mV/s. The weld sample was ultrasonically cleaned with alcohol for 30 min after the electrochemical test. The microstructure of the electrochemical corrosion behavior was analyzed by scanning electron microscopy.

## 3. Result and Discussion

### 3.1. Weld Surface Quality

**Figure 3:**
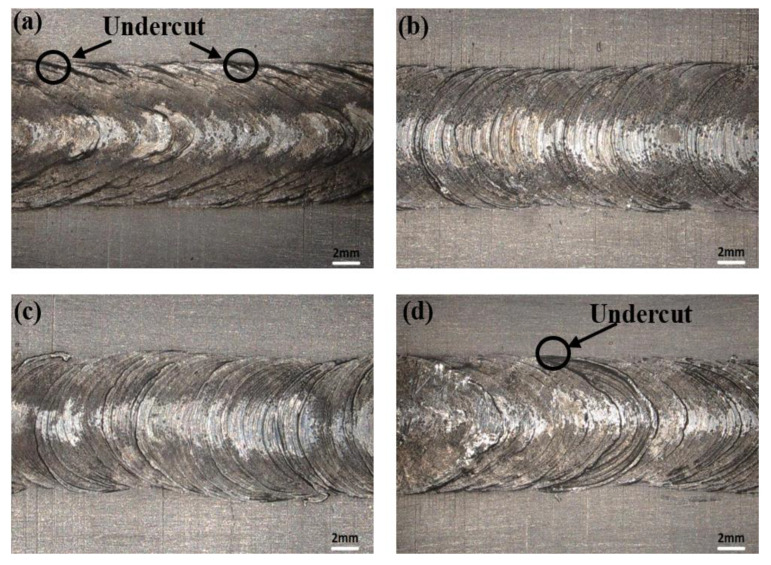
The morphology of weld surface: (**a**) 0 W; (**b**) 60 W; (**c**) 80 W; (**d**) 100 W.

The surface morphologies of welds produced by ultrasonic assistance are shown in Figure 3. It can be observed from Figure 3a that the undercut occurs on both sides of the weld seam, due to excessively rapid cooling at the edges of the molten pool in the absence of ultrasonic vibration. With the ultrasonic power adjusted to 60 W and 80 W, the defects on the weld surface tended to decrease. The addition of ultrasonic vibration assistance enhanced the fluidity of the liquid metal and accelerated heat transfer. This effect reduced the temperature gradient between the edge and the center laser molten pool while making the weld surface smoother and uniform. With the ultrasonic power increased to 100 W, the stability of the laser keyhole was disrupted due to a violent cavitation effect, which led to gas entrapment and pore formation. Furthermore, the overpowered acoustic streaming caused turbulent flow in the molten pool, resulting in surface irregularities and undercuts, as shown in Figure 3d.

**Figure 4:**
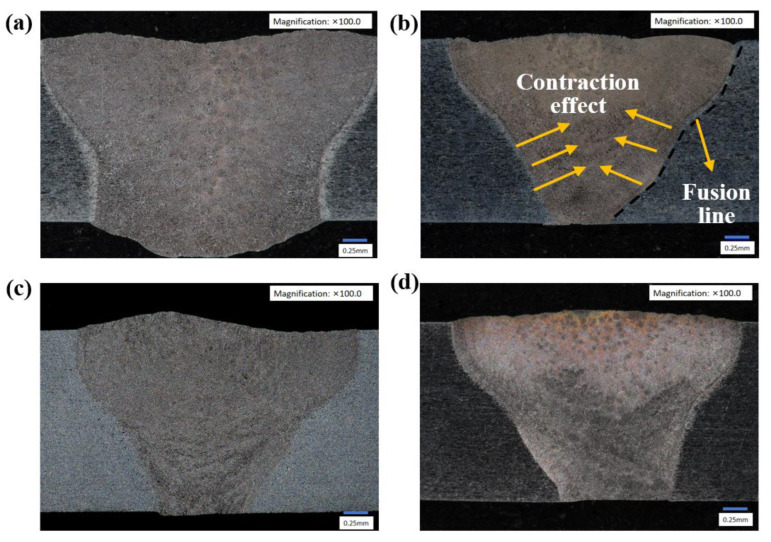
Weld cross-section morphology: (**a**) 0 W; (**b**) 60 W; (**c**) 80 W; (**d**) 100 W.

The cross-sectional morphology of the weld seam is shown in Figure 4. By comparing Figure 4a,c, it can be observed that the cross-section of the weld seam revealed a significant size contraction effect after the addition of ultrasonic. The surface weld width decreased from 3.50 mm to 2.87 mm, and the root width decreased from 2.37 mm to 0.90 mm. The ultrasonic-induced acoustic streaming effect generated centripetal vortex flows within the molten pool, which drove the liquid metal toward the weld centerline and resulted in molten pool contraction with significant width reduction, as exhibited in Figure 4b. The weld root direct contact with the ultrasonic horn markedly increased metal fluidity, leading to a more pronounced contraction effect.

**Figure 5:**
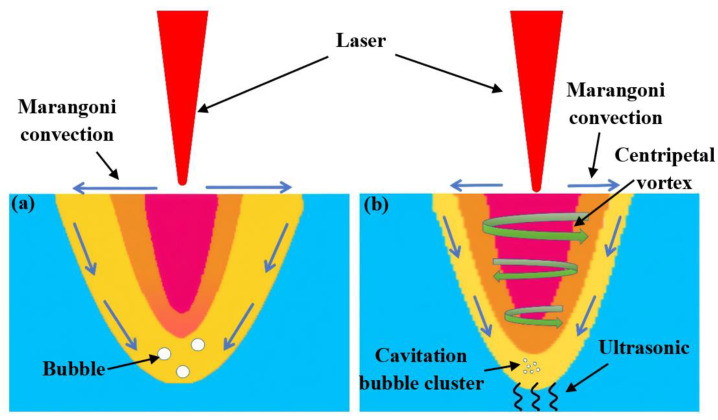
Molten pool behavior in laser welding: (**a**) No ultrasonic; (**b**) Ultrasonic.

The application of ultrasonic vibrations can alter the flow characteristics and temperature gradient of the laser welding molten pool. The conventional laser welding causes molten pool flow primarily governed by the Marangoni effect, as shown in Figure 5a [21]. This generated surface flow from the center of the high temperature area to the edge of the low temperature area, resulting in large temperature gradients and promoting defect formation. The acoustic streaming effect generated by the ultrasound interacted with the Marangoni convection, thereby altering the flow pattern of the molten pool, as shown in Figure 5b. The formed centripetal vortex enhanced heat transfer within the molten pool and improved temperature uniformity [22]. The acoustic streaming-induced stirring effect attenuated the Marangoni convection, resulting in molten pool contraction toward the central region and consequent narrowing of the weld cross-section. The ultrasonic cavitation effect promoted bubble collapse, thereby accelerating heat dissipation during molten pool solidification.

### 3.2. Microstructural Characterization

**Figure 6:**
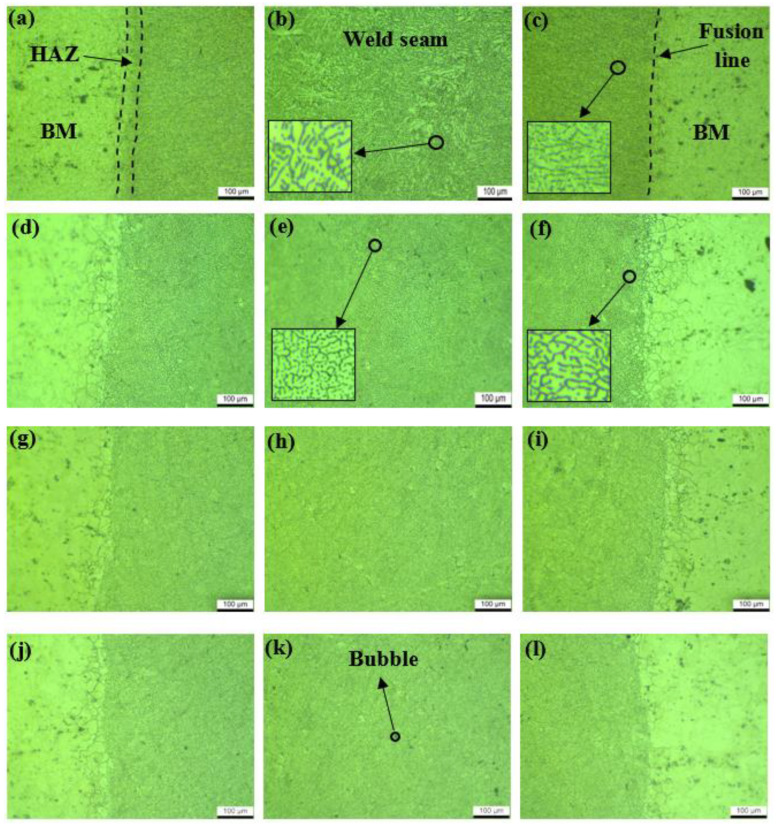
Microstructure morphology of welds: (**a**–**c**) 0 W; (**d**–**f**) 60 W; (**g**–**i**) 80 W; (**j**–**l**) 100 W.

The microstructure of the weld seam is shown in Figure 6. The weld center part exhibited equiaxed grain characteristics, while columnar grain dominated the edge regions. The HAZ was a characteristic region located between the fusion line and the base metal. It served as a transitional microstructural zone from the base metal to the weld. The sample without ultrasonic assistance exhibited heterogeneous grain size distribution in Figure 6b. The fusion line region exhibited coarse columnar grains with pronounced growth orientation toward the weld center, as shown in Figure 6c. In contrast, with ultrasonic assistance, the columnar grain around the fusion line transformed into equiaxed grain and the interdendritic spacing decreased significantly in Figure 6f. The refined grain was observed in the weld center and the size is reduced by about 40% compared to when no ultrasound is applied, along with reduced width of the heat-affected zone in Figure 6d,e. With the ultrasonic power increased to 80 W, the transition zone between the base metal and weld seam exhibits significant uniformity in Figure 6g–i. The acoustic pressure gradient formed by energy dissipation during ultrasonic propagation can enhance the plastic flow of metals and exert a strong stirring effect on high-viscosity medium [23]. The tiny weld bubbles observed at 100 W ultrasonic power are shown in Figure 6k. The formation of bubbles was attributed to the ultrasonic cavitation intensity that surpasses an optimal threshold. The violent collapse of cavitation bubbles generates shock waves that disrupt the stability of the laser keyhole. This instability causes the keyhole to fluctuate or collapse intermittently, readily trapping gas bubbles and leading to the formation of macro-pores [24].

**Figure 7:**
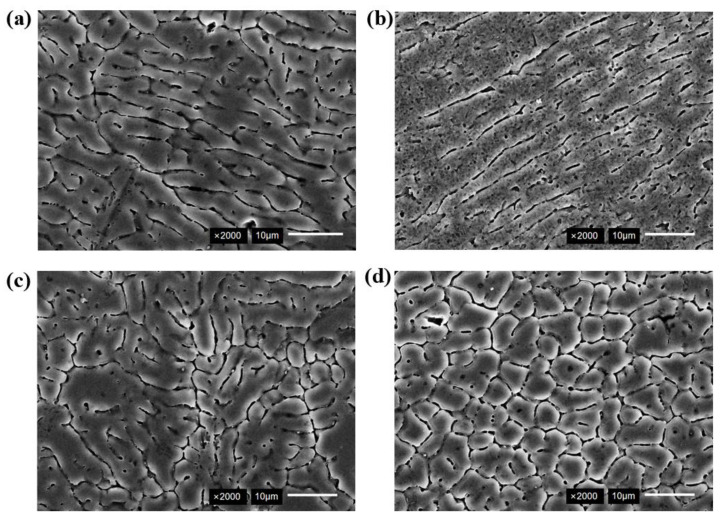
Fusion boundary zone: (**a**) no ultrasonic; (**b**) ultrasonic; (**c**) no ultrasonic; (**d**) ultrasonic.

**Figure 8:**
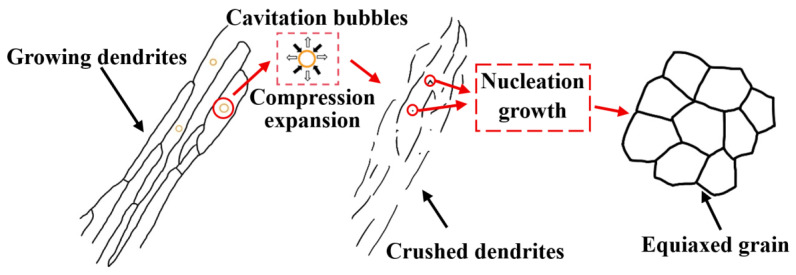
Schematic diagram of ultrasonic dendrites refinement effect.

The metallographic structure of different zones in the weld joint was shown in Figure 7. The typical columnar crystal structures were observed near the fusion line, as shown in Figure 7a. The dendrite arms exhibited relative continuity with individual precipitation lengths reaching up to 25 μm. The metallographic structure of the fusion line region after ultrasonic vibration is shown in Figure 7b. The columnar crystal structure was fragmented by the cavitation effect of ultrasonic, with dendrites fractured at multiple locations. The dendritic arm was relatively dispersed and continuity was reduced, as shown in Figure 7b. The metallographic structure of the weld center is presented in Figure 7c,d. The equiaxed grains were refined and distributed uniformly after ultrasonic vibration in Figure 7d. The grain refinement mechanism is shown in Figure 8. The cavitation bubbles in the liquid vibrated under ultrasonic action, and the dynamic process of growth and collapse occurred as acoustic pressure reached specific threshold values. The high-speed collapse of these cavitation bubbles generated instantaneous high temperature and high pressure in the surrounding molten area. The instantaneous temperature and pressure increased the solidification point of the alloy and enhanced the condensate pressure drop of the melt, thereby promoting nucleation. Additionally, high temperatures generated by cavitation induced grain coalescence and increased the number of nucleation cores, thus increased the number of grains [25].

### 3.3. Polarization Curve and Impedance Spectroscopy Analysis

**Figure 9:**
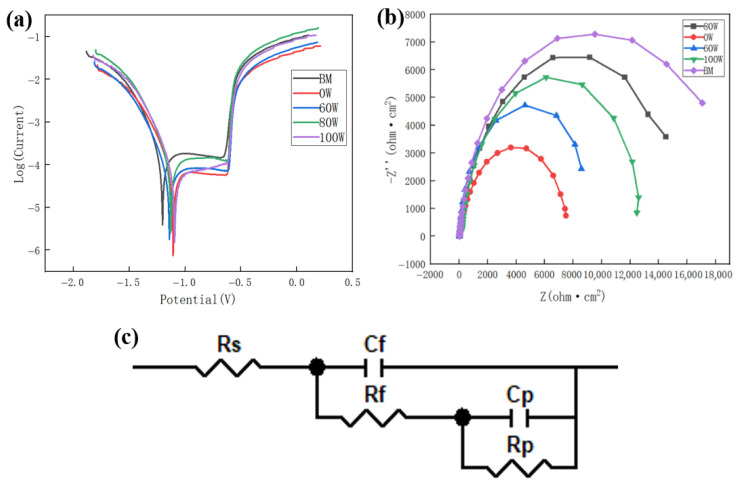
Electrochemical test: (**a**) electrochemical polarization curve; (**b**) Nyquist plots; (**c**) equivalent circuit.

The potentiodynamic polarization curves of each welded joint in 3.5% NaCl solution were shown in Figure 9a. The corrosion characteristics of all specimens exhibited consistent behavior with notable passive regions. The critical time for pitting initiation was progressively delayed with increasing ultrasonic power, indicating enhanced stability of the passive film characteristic. The electrochemical fitting results are presented in Table 3. *I_corr_* served as the key evaluation criterion for corrosion resistance, exhibiting direct proportionality to corrosion rate. According to Table 3, the *I_corr_* of the base metal sample was 2.688 × 10^−5^ A·cm^−2^. The *I_corr_* of the sample without ultrasonic treatment was 4.511 × 10^−5^ A·cm^−2^. The *I_corr_* reached a minimum value of 3.350 × 10^−5^ A·cm^−2^ when the ultrasonic power was adjusted to 80 W. The *I_corr_* increased to 3.937 × 10^−5^ A·cm^−2^ as the ultrasonic power was increased to 100 W. When the ultrasonic power increased from 0 W to 80 W, the grain refinement effect of the ultrasound gradually strengthened. The increased number of grain boundaries per unit area led to more tortuous corrosion paths, resulting in improved corrosion resistance. However, when the ultrasonic power was further increased to 100 W, the higher power caused instability within the molten pool, generating a greater number of pores [24]. These pores acted as initiation sites for corrosion, thereby reducing the corrosion resistance. The optimal corrosion resistance was achieved at 80 W, reaching 81.8% of the base metal, representing a 19.09% improvement compared to the no-ultrasonic sample.

The Nyquist plots for each sample, as shown in Figure 9b. The equivalent circuit shown in Figure 9c was employed to characterize the corrosion behavior of the joint [26]. The polarization resistance *Rp* of alloys was inversely proportionality related with corrosion rate [27,28]. The polarization resistance increased from 7897 Ω·cm^−2^ to 12,628 Ω·cm^−2^ as ultrasonic power increased from 0 W to 80 W, as shown in Table 3. The polarization resistance decreased to 9078 Ω·cm^−2^ when ultrasonic power was increased to 100 W. The evolution trend of corrosion resistance agreed with the result obtained from the polarization curves as shown in Figure 9a.

### 3.4. Local Corrosion Behavior

**Figure 10:**
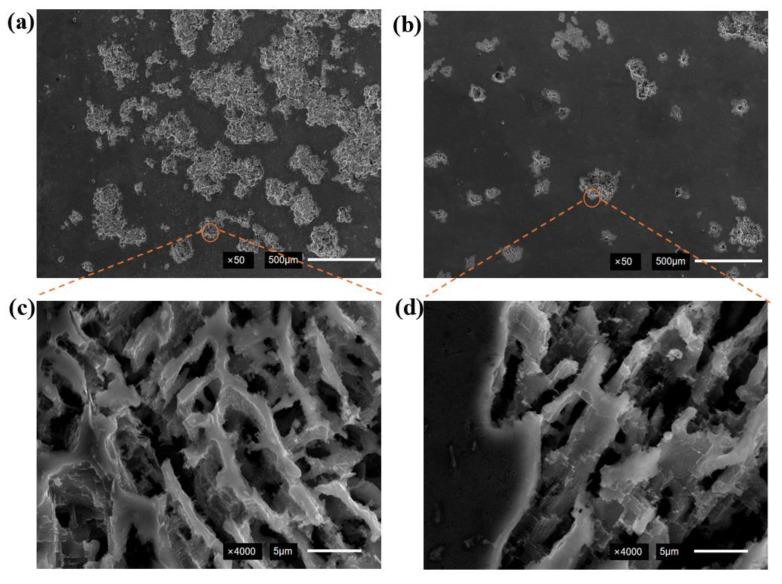
Corrosion morphology of SEM: (**a**) no ultrasonic; (**b**) ultrasonic; (**c**) no ultrasonic; (**d**) ultrasonic.

The corrosion morphology after the electrochemical test is shown in Figure 10. The specimen without ultrasonic treatment exhibited larger corrosion pit areas with continuous distribution, as shown in Figure 10a. The corrosion area of the weld after ultrasonic assistance was reduced by approximately 62% compared to that without ultrasonic treatment, as shown in Figure 10b. The corrosion propagation along interdendritic pathway, which formed a continuous network of grooves, is shown in Figure 10c. The dendritic structures were fragmented and reduced their continuity after ultrasonic assistance. The penetration pathways for corrosive media were obstructed, leading to the formation of uniform shallow corrosion in Figure 10d.

**Figure 11:**
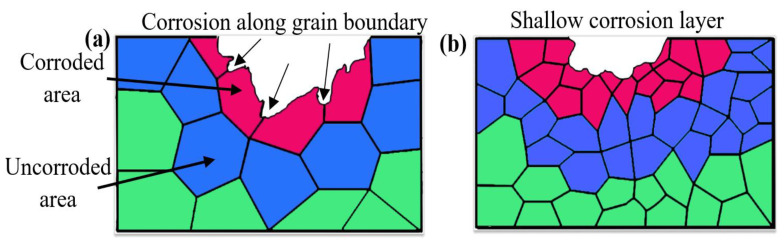
Schematic diagram of dendrite structure-induced corrosion mechanism: (**a**) no ultrasonic; (**b**) ultrasonic.

The mechanism of dendrite-induced corrosion is shown in Figure 11. The larger grain sizes were observed under no ultrasonic treatment, with corrosive media propagating along dendrites to form deeper corrosion pits, as shown in Figure 11a. The ultrasonic assistance resulted in grain refinement with increased grain boundary density per unit area, as shown in Figure 11b. The corrosion propagation along grain boundaries was repeatedly deflected by refined grain structures, thereby decreased corrosion rates. The large intergranular corrosion pits were transformed into shallow intergranular corrosion layers.

### 3.5. Mechanical Performance Analysis

**Figure 12:**
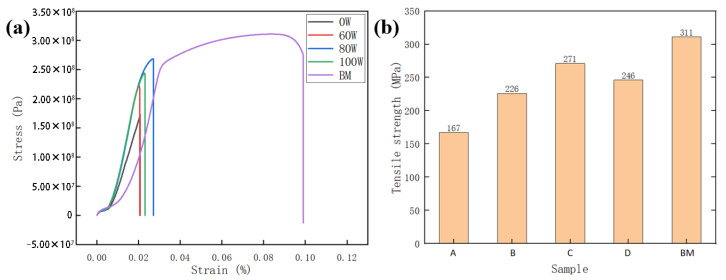
Tensile test data: (**a**) stretching curve; (**b**) tensile strength.

The tensile test results of the samples are shown in Figure 12. The fracture location in all specimens was in the weld zone. The tensile strength improved as ultrasonic power increased from 0 W to 80 W, as shown in Figure 12b. When the ultrasonic power increased to 100 W, the excessive power resulted in the formation of large pores in the molten pool. The pores acted as typical stress concentration points, from which cracks preferentially initiated and propagated rapidly during tensile testing, leading to a decrease in tensile strength. The tensile strength of the joint without ultrasonic treatment was 167 MPa. The tensile strength reached 271 MPa at the adjusted ultrasonic power of 80 W, which was 62% higher than that with no ultrasonic treatment, and reached 87% of the base metal. The grain refinement effect induced by ultrasonic assistance enhanced weld joint tensile properties.

**Figure 13:**
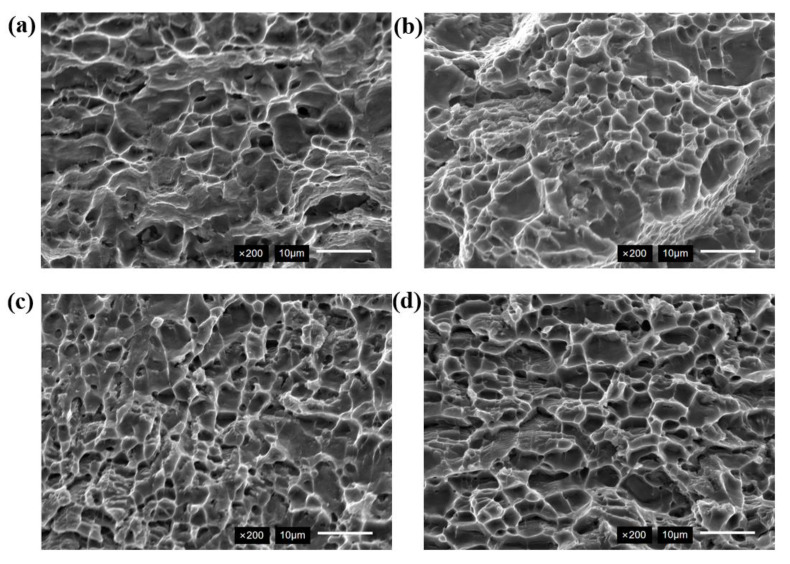
Tensile fracture morphology (**a**) 0 W; (**b**) 60 W; (**c**) 80 W; (**d**) 100 W.

The tensile fracture morphology is shown in Figure 13. The tensile fracture mode of the sample without ultrasonic treatment was a combination of ductile fracture and quasi-cleavage fracture. The dimple exhibited shallow depth and non-uniform size distribution, as shown in Figure 13a. The dimples displayed directional alignment characteristic of tearing dimples. The porosity in the weld zone induced stress concentration. The formation was primarily caused by porosity in the weld, which resulted in stress concentration. In the presence of a larger external force, the crack initially formed at the porosity, and the zone that is affected by the tearing stress around the crack finally formed the tear dimple. The dimple remained scarce even at 60 W ultrasonic power, as shown in Figure 13b. The tensile fracture surface exhibited typical ductile fracture characteristics when ultrasonic power exceeded 60 W. The dimple density increased with refined size distribution and significant depth in Figure 13c,d.

## 4. Conclusions

This study demonstrates that ultrasonic assistance is an effective strategy for simultaneously enhancing the mechanical properties and corrosion resistance of 6061-T6 aluminum alloy laser welds. The main findings and their implications are as follows:

1. The dominant acoustic streaming effect was found to alter the molten pool flow dynamics, overriding the Marangoni convection and resulting in a narrower weld with reduced undercut.
2. The key mechanism for property enhancement is ultrasonic cavitation, which effectively fragments dendritic crystals and promotes homogeneous equiaxed grain formation. This microstructural refinement is identified as the primary factor in blocking corrosion penetration paths.
3. The area and number of corrosion pits decreased with ultrasonic assistance. The optimal corrosion resistance was achieved at 80 W ultrasonic power, representing a 19.09% enhancement compared to ultrasonic treatment. This quantifies the substantial benefit of ultrasound for applications in corrosive environments.
4. The grain refinement effect of ultrasonic treatment improved the tensile strength of the welded joint, while dimples became denser and deeper. This demonstrates the potential of ultrasonic assistance to produce welded joints that meet or exceed the performance requirements of critical lightweight structures.

## Figures and Tables

**Table 1:** Chemical composition of the 6061-T6 aluminum alloy.

Element	Si	Cu	Fe	Mn	Zn	Mg	Cr	Ti	Al
Content/%	0.72	0.36	0.70	0.15	0.25	0.93	0.32	0.15	Bal

**Table 2:** Welding parameters.

Sample Number	Laser Power/W	Welding Speed/m·s^−1^	Defocus Amount/mm	Ultrasonic Power/W
A	2800	0.03	0.4	0
B	2800	0.03	0.4	60
C	2800	0.03	0.4	80
D	2800	0.03	0.4	100

**Table 3:** Electrochemical fitting results of weld joint.

Sample	*I_corr_*/A·cm^−2^	*E_co_*_rr_/V	*R_p_*/Ω·cm^−2^
BM	2.688 × 10^−5^	−1.20	14,938
A	4.511 × 10^−5^	−1.06	7897
B	4.021 × 10^−5^	−1.13	9044
C	3.650 × 10^−5^	−1.11	12,628
D	3.988 × 10^−5^	−1.04	9078

## Data Availability

The original contributions presented in this study are included in the article. Further inquiries can be directed to the corresponding author.
